# Dietary-challenged mice with Alzheimer-like pathology show increased energy expenditure and reduced adipocyte hypertrophy and steatosis

**DOI:** 10.18632/aging.202978

**Published:** 2021-04-16

**Authors:** Stefanie Schreyer, Nikolaus Berndt, Johannes Eckstein, Michael Mülleder, Shabnam Hemmati-Sadeghi, Charlotte Klein, Basim Abuelnor, Alina Panzel, David Meierhofer, Joachim Spranger, Barbara Steiner, Sebastian Brachs

**Affiliations:** 1 Department of Neurology, Charité – Universitätsmedizin Berlin, Corporate Member of Freie Universität Berlin and Humboldt-Universität zu Berlin, Berlin 10117, Germany; 2 Institute for Imaging Science and Computational Modelling in Cardiovascular Medicine, Charité – Universitätsmedizin Berlin, Corporate Member of Freie Universität Berlin and Humboldt-Universität zu Berlin, Berlin 13353, Germany; 3 Institute of Biochemistry, Charité – Universitätsmedizin Berlin, Corporate Member of Freie Universität Berlin and Humboldt-Universität zu Berlin, Berlin 10117, Germany; 4 Core Facility High Throughput Mass Spectrometry, Charité – Universitätsmedizin Berlin, Corporate Member of Freie Universität Berlin and Humboldt-Universität zu Berlin, Berlin 10117, Germany; 5 Max Planck Institute for Molecular Genetics, Berlin 14195, Germany; 6 Department of Endocrinology and Metabolism, Charité – Universitätsmedizin Berlin, Corporate Member of Freie Universität Berlin and Humboldt-Universität zu Berlin, Berlin 10117, Germany; 7 DZHK (German Centre for Cardiovascular Research), Partner Site Berlin, Berlin, Germany

**Keywords:** Alzheimer's disease, diet-induced obesity, hypertrophy, energy expenditure, steatosis

## Abstract

Alzheimer’s disease (AD) is frequently accompanied by progressing weight loss, correlating with mortality. Counter-intuitively, weight loss in old age might predict AD onset but obesity in midlife increases AD risk. Furthermore, AD is associated with diabetes-like alterations in glucose metabolism. Here, we investigated metabolic features of amyloid precursor protein overexpressing APP23 female mice modeling AD upon long-term challenge with high-sucrose (HSD) or high-fat diet (HFD). Compared to wild type littermates (WT), APP23 females were less prone to mild HSD-induced and considerable HFD-induced glucose tolerance deterioration, despite unaltered glucose tolerance during normal-control diet. Indirect calorimetry revealed increased energy expenditure and hyperactivity in APP23 females. Dietary interventions, especially HFD, had weaker effects on lean and fat mass gain, steatosis and adipocyte hypertrophy of APP23 than WT mice, as shown by ^1^H-magnetic-resonance-spectroscopy, histological and biochemical analyses. Proteome analysis revealed differentially regulated expression of mitochondrial proteins in APP23 livers and brains. In conclusion, hyperactivity, increased metabolic rate, and global mitochondrial dysfunction potentially add up to the development of AD-related body weight changes in APP23 females, becoming especially evident during diet-induced metabolic challenge. These findings emphasize the importance of translating this metabolic phenotyping into human research to decode the metabolic component in AD pathogenesis.

## INTRODUCTION

To date, Alzheimer’s disease (AD) cannot be cured and its underlying mechanisms remain elusive [1]. Early diagnosis of AD – a crucial requirement for the application of existing symptomatic treatment – is complicated due to its multifaceted pathology and a variety of accessory symptoms, which are partially not well understood [2]. Thus, basic research focusing on these symptoms might provide important insights into pathological mechanisms leading to new approaches for diagnosis and therapy.

Worldwide, about 50 million patients suffer from AD, the most common neurodegenerative disease [3]. Brains affected by AD are characterized by extracellular amyloid-beta (Aβ) aggregates, intracellular neurofibrillary tangles of hyperphosphorylated tau, and progressive neurodegeneration [4, 5]. Synapse and neuronal loss ultimately result in dementia [6]. For decades, AD research focused on Aβ – cleaved from the amyloid precursor protein (APP) – and its aggregation into plaques as disease etiology [7]. Nevertheless, decreasing Aβ burden using antibodies has failed to significantly improve patients’ condition [8]. Thus, alternative causes came into the spotlight: Epidemiologic studies identified metabolic dysfunctions such as insulin resistance and glucose insensitivity as risk factors, potentially involved in AD pathology [9, 10]. These shared features lead to the suggestion that AD might represent brain-specific diabetes [11]. However, the exact mechanisms linking both pathologies remain unknown.

Counter-intuitively, both obesity in midlife and low body weight in old age increase the risk of developing AD [12, 13]. However, at least 30-40% of patients suffer from AD-related involuntary weight loss, aggravating as AD progresses leading to poorer health, reduced quality of life, and increased mortality [14]. Since weight loss already occurs years before the onset of clinical AD symptoms, body weight changes in old age might represent early AD manifestations potentially contributing to early diagnosis [15, 16].

Weight loss occurs with negative energy balance, i.e. lower energy intake than energy expenditure [17]. Reasons for negative energy balance might be reduced energy intake, e.g. due to reduced food intake or malabsorption [18], hypermetabolism originating from elevated resting energy expenditure, e.g. increased thermogenesis, or elevated total energy expenditure, e.g. increased physical activity [18]. Previous body weight analyses in AD patients remained inconclusive, requiring further research to elucidate underlying mechanisms of AD-related weight loss.

Here, we investigate early AD-related metabolism and body weight changes challenging the APP-overexpressing APP23 mouse model [19] with different diets before Aβ-plaque development to extensively characterize its metabolic features and the impact of different fuel sources. Thereby, we aim to gain insights into underlying mechanisms of AD-related metabolic and body weight changes, which might provide valuable information for early diagnosis or therapeutic approaches.

## RESULTS

### APP23 mice showed lower body weight due to lower fat and lean mass

**Figure 1:**
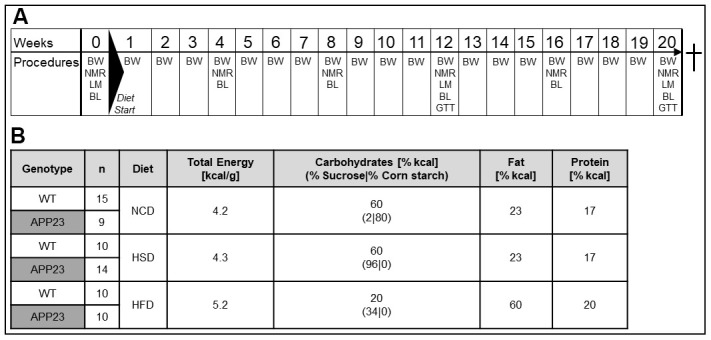
**Experimental design and nutritional values of diets.** (**A**) Experimental procedures: In week 0, baseline measurements of body composition (NMR) and indirect calorimetry (LM) were conducted, as well as the first blood withdrawal (BL). Diets were fed from week 1 to 20. Body weight was assessed weekly. Monthly, NMR and BL were performed. In week 12 and 20, mice additionally underwent LM measurements and glucose tolerance tests (GTT). (**B**) Group layout and diet composition: 4-6-week old transgenic APP23 and WT mice were assigned to either normal-control diet (NCD), high-sucrose diet (HSD) or high-fat diet (HFD). Shown n numbers represent animal numbers for all measurements in living animals and in the animals’ blood.

**Figure 2:**
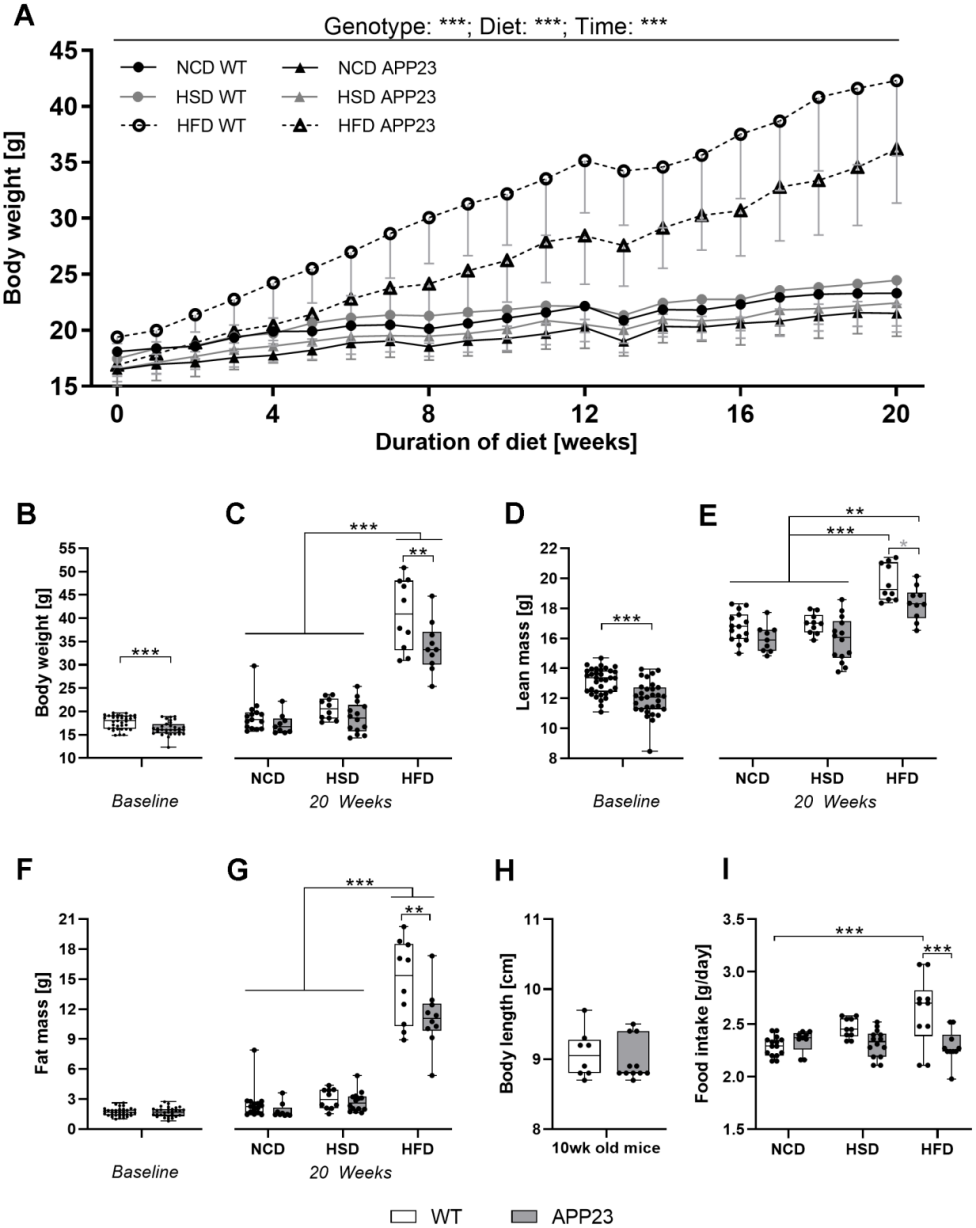
**Body weight, body composition, body length, and food intake.** (**A**) Development of body weight during 20 weeks of dietary intervention (NCD, HSD or HFD). (**B**) Body weight at baseline (week 0). (**C**) Body weight after 20 weeks of dietary intervention. (**D**) Lean mass at baseline (week 0). (**E**) Lean mass after 20 weeks of dietary intervention. (**F**) Fat mass at baseline (week 0). (**G**) Fat mass after 20 weeks of dietary intervention. (**H**) Exemplarily measured body length in male and female adult (mean age 10 weeks) APP23 and WT mice. (**I**) Mean daily food intake averaged over the entire intervention and across mice occupying the same cage. Data are represented as box (25^th^ to 75^th^ percentile) with median and whiskers from minimum to maximum. Black asterisks indicate significant differences between groups (*: p<0.05; **: p<0.01; ***: p<0.001), gray asterisk indicates a statistical trend towards significance (p<0.1) according to nonparametric ANOVA-type statistics (**A**), ordinary 2-way ANOVA with Tukey post-hoc test (**C**, **G**, **I**), multiple contrast Tukey-type test (**E**), or nonparametric t-test (**B**, **D**, **F**, **H**). n_Week 0 WT_=35, n_Week 0 APP23_=31, n_NCD WT_=15, n_NCD APP23_=9, n_HSD WT_=10, n_HSD APP23_=14, n_HFD WT_=10, n_HFD APP23_=10; for body length: n_WT_=8, n_APP23_=11.

To investigate the impact of APP overexpression on metabolism, APP23 and WT mice were challenged with normal-control (NCD), high-sucrose (HSD) or high-fat diet (HFD) for 20 weeks (Figure 1A, 1B). Across the experiment, all groups significantly gained body weight (F(4.04,52.55)=484.37, p<0.001; Figure 2A). However, APP23 mice showed a significantly lower body weight (F(1.00,52.55)=30.25, p<0.001). The effect of diet (F(1.90,52.55)=63.47, p<0.001) was most prominent in HFD (up to 41% higher body weight compared to NCD/HSD), while HSD had virtually no effect. At baseline, body weight of APP23 mice was 8% lower compared to WT mice (p=0.001; Figure 2B). This body weight difference remained constant until week 20 in NCD- and HSD-fed mice (NCD: 6%, n.s.; HSD: 7%, n.s.; Figure 2C). After 20 weeks of dietary intervention, HFD elevated body weight up to 85% in WT mice and up to 68% in APP23 mice (both p<0.001), while HSD had no effect on body weight. The body weight difference between genotypes rose to 15% in HFD-fed mice (p=0.008).

Lean mass was 10% lower in APP23 mice at baseline (week 0, p<0.001; Figure 2D). After 20 weeks of dietary intervention, lean mass was up to 7% lower in NCD- and HSD-fed APP23 mice, however not significant, and 8% lower in HFD-fed APP23 mice (p=0.087; Figure 2E). While HSD did not affect lean mass, HFD significantly increased lean mass in both genotypes but more prominently in WT mice (HFD WT vs. NCD/HSD WT, both p<0.001; HFD APP23 vs. NCD/HSD APP23, both p≤0.005). Fat mass was equal between genotypes at baseline (week 0; Figure 2F). While 20 weeks of HSD had almost no effect on fat mass, HFD significantly elevated fat mass in both genotypes (p<0.001; Figure 2G). Fat mass was 37% lower in HFD-fed APP23 mice (p=0.007) and up to 10% lower during NCD and HSD, however not significant. Lower body weight did not originate from a smaller growth as body length was almost identical in young adult APP23 and WT mice (Figure 2H). Notably, food intake gradually increased in WT mice from NCD to HSD to HFD (NCD WT vs. HFD WT, p<0.001), whereas it remained constant between diets in APP23 mice resulting in a significantly lower HFD intake compared to WT mice (p<0.001; Figure 2I).

In summary, APP23 mice showed lower body weight due to both lower fat and lean mass. The difference in lean mass was already present at baseline, whereas the difference in fat mass occurred only at the end of dietary intervention. APP23 mice could not catch up for this body weight difference due to a similar body weight gain.

### Diet-induced adipocyte hypertrophy and steatosis were extenuated in APP23 mice

**Figure 3:**
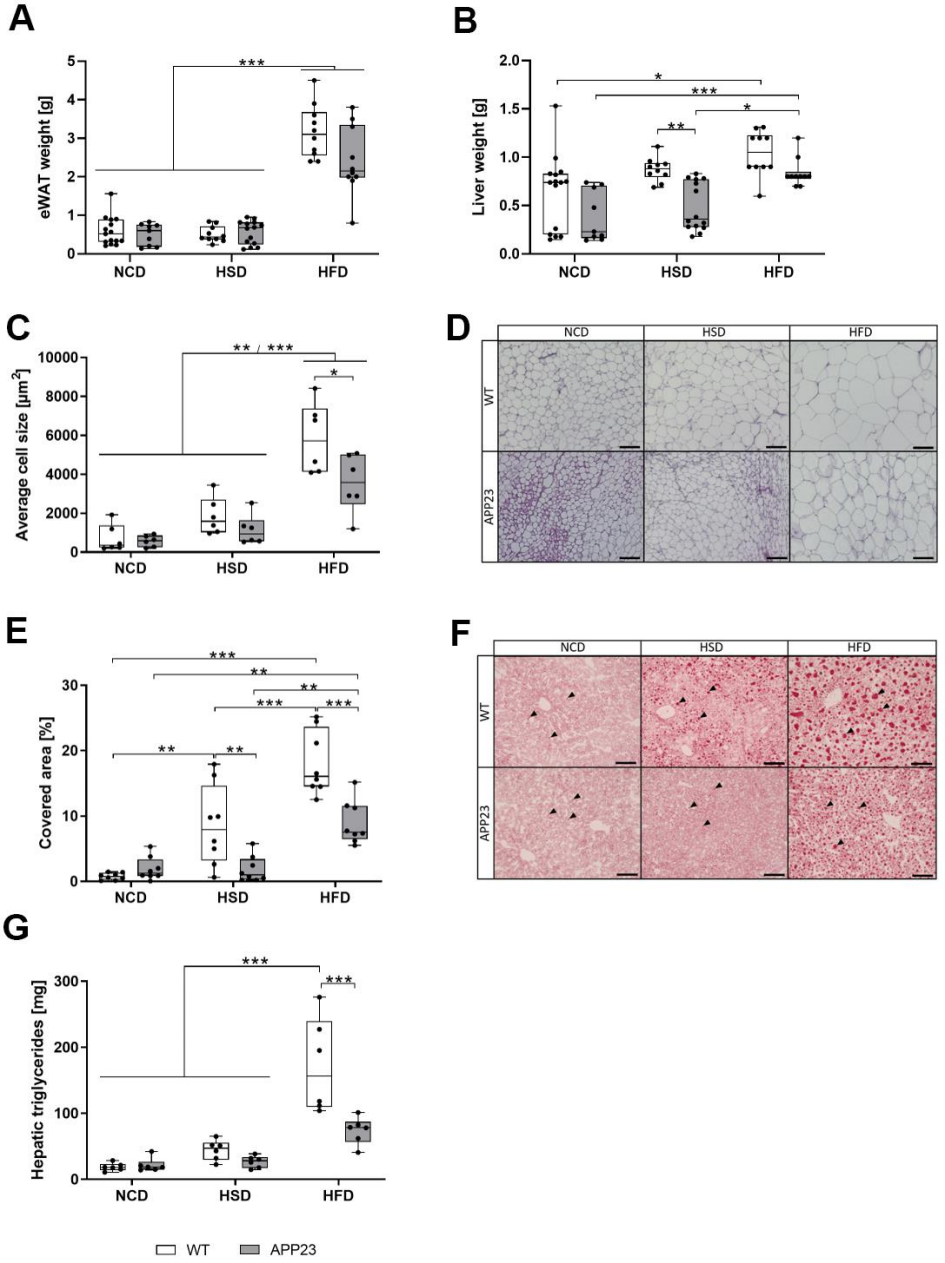
**Weight of eWAT and liver, histological analysis of adipocyte size in eWAT and lipid droplets in liver tissue, and quantification of hepatic triglycerides.** Weight of eWAT (**A**) and liver (**B**) upon sacrifice of mice after 20 weeks of dietary interventions (NCD, HSD or HFD). (**C**) Quantification of mean adipocyte size in eWAT sections analyzed with ImageJ (**D**) Representative hematoxylin/eosin stainings of eWAT tissue. Scale bar: 100 μm (**E**) Lipid quantification of Oil Red O-stained hepatic sections analyzed with ImageJ. (**F**) Representative Oil Red O stainings of liver tissue. Arrowheads point to individual Oil Red O-stained lipid droplets. Scale bar: 100 μm. (**G**) Quantification of hepatic triglycerides. Measured triglyceride concentrations were multiplied by liver weight to obtain absolute amounts of triglycerides. Data are represented as box (25^th^ to 75^th^ percentile) with median and whiskers from minimum to maximum. Black asterisks indicate significant differences between groups (*: p<0.05; **: p<0.01; ***: p<0.001), gray asterisk indicates a statistical trend towards significance (p<0.1) according to nonparametric multiple contrast Tukey-type test (**A**, **B**) and Tukey post-hoc test of an ordinary 2-way ANOVA (**C**, **E**, **G**). For (**A**, **B**) n_NCD WT_=15, n_NCD APP23_=9, n_HSD WT_=10, n_HSD APP23_=14, n_HFD WT_=10, n_HFD APP23_=10. For (**C**, **D**) n=6 each. For (**E**, **F**) n=8 each, For (**G**) n=6 each.

To further examine body composition changes, tissues were analyzed upon sacrifice. Epigonadal white adipose tissue (eWAT) weight was similar between genotypes and only elevated by HFD (5-fold, vs. NCD/HSD each p<0.001; Figure 3A). Liver weight was also similar during NCD and elevated by HFD (e.g. NCD APP23 vs. HFD APP23, p<0.001) but 45% (p=0.002) and 20% (n.s.) lower in HSD- and HFD-fed APP23 mice (Figure 3B). Adipocyte size was analyzed in hematoxylin/eosin-stained eWAT (Figure 3C, 3D). APP23 mice displayed 20% (NCD, n.s.) to 40% (HSD, n.s.; HFD, p=0.015) smaller adipocytes than WT mice. In contrast to unaffected eWAT weight, HSD increased adipocyte size 2.3-fold (n.s.). HFD rose adipocyte size compared to NCD (7.3-fold, NCD WT/APP23 vs. HFD WT/APP23, both p<0.001) and HSD (3.1-fold, HSD WT vs. HFD WT, p<0.001; HSD APP23 vs. HFD APP23, p=0.010). Moreover, hepatic fat content (i.e. liver steatosis) was analyzed by Oil Red O staining and triglyceride quantification (Figure 3E–3G). During NCD, lipid droplet size and amount was similar, while HSD- and HFD-fed APP23 mice embodied less and smaller droplets than WT mice (HSD: 4.5-fold, p=0.009; HFD: 2-fold, p<0.001; Figure 3E, 3F). According to increasing liver weight, lipid droplet size and amount gradually rose from NCD to HFD, specifically prominent in WT mice (NCD vs. HSD: 11-fold, p=0.002; HSD vs. HFD: 2-fold, p<0.001). Furthermore, hepatic triglycerides were similar between genotypes and diets during NCD and HSD (Figure 3G). In contrast, HFD-fed WT mice showed up to 9.2-fold elevated hepatic triglycerides compared to NCD- and HSD-fed mice of both genotypes (p<0.001), whereas triglycerides in HFD-fed APP23 mice were only increased by 3.4-fold (n.s.). Thus, during HFD hepatic triglycerides were 2.3-fold higher in WT mice compared to APP23 mice (p<0.001).

Altogether, APP23 mice resisted against HSD- and HFD-induced hepatic weight gain, lipid accumulation and adipocyte hypertrophy.

### Insulin levels were unaltered in APP23 mice during NCD and HSD but lower during HFD

**Figure 4:**
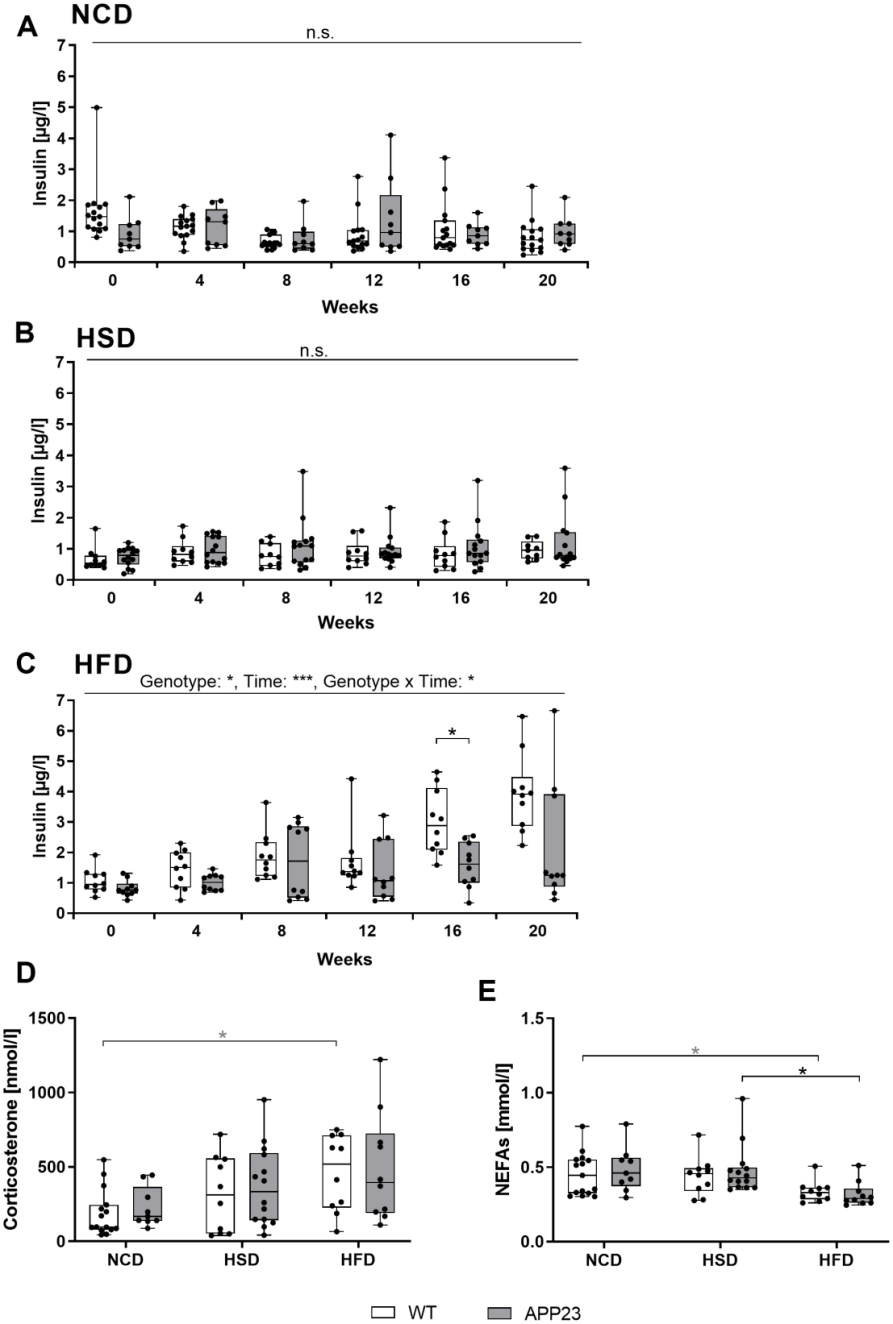
**Analysis of basic metabolic parameters in blood of fed mice.** (**A**–**C**) Plasma insulin measured in NCD-fed (**A**), HSD-fed (**B**) and HFD-fed (**C**) mice at baseline (week 0) and after 4, 8, 12, 16, and 20 weeks of diet. Corticosterone levels (**D**) and NEFA levels (**E**) measured in blood plasma obtained upon sacrifice after 20 weeks of diet. Data are represented as box (25^th^ to 75^th^ percentile) with median and whiskers from minimum to maximum. Black asterisks indicate significant differences between groups (*: p<0.05; **: p<0.01; ***: p<0.001), gray asterisk indicates a statistical trend towards significance (p<0.1) according to Tukey post-hoc test of repeated measures ANOVA (**A**–**C**) and nonparametric multiple contrast Tukey-type test (**D**, **E**). n_NCD WT_=15, n_NCD APP23_=9, n_HSD WT_=10, n_HSD APP23_=14, n_HFD WT_=10, n_HFD APP23_=10.

To investigate glucose homeostasis, we monthly analyzed plasma insulin levels in fed mice (Figure 4). NCD and HSD did not alter insulin levels, with APP23 mice showing no differences compared to WT mice (Figure 4A, 4B). In contrast, HFD elevated insulin levels over time (F(2.75,49.50)=15.72, p<0.001), starting around week 8 of dietary intervention (Figure 4C). Interestingly, genotypes were differentially affected by the HFD-induced elevation of insulin levels (F(1,8)=6.915, p=0.017). In week 16 (p=0.019) and 20 (n.s., p=0.182), insulin levels of WT mice were almost twice as high as those of APP23 mice. In all, only HFD elevated insulin levels, whereas APP23 mice were less affected. Additionally, corticosterone and non-esterified free fatty acid (NEFA) levels were measured in final plasma. Corticosterone levels were similar between genotypes and increased by trend from NCD to HFD (NCD WT vs. HFD WT, p=0.098) (Figure 4D). In contrast, NEFA levels slightly decreased during HFD but similarly between genotypes (NCD APP23 vs. HFD APP23, p=0.062; HSD APP23 vs. HFD APP23, p=0.023; Figure 4E).

### HFD deteriorated glucose tolerance earlier and stronger than HSD but less in APP23 mice

**Figure 5:**
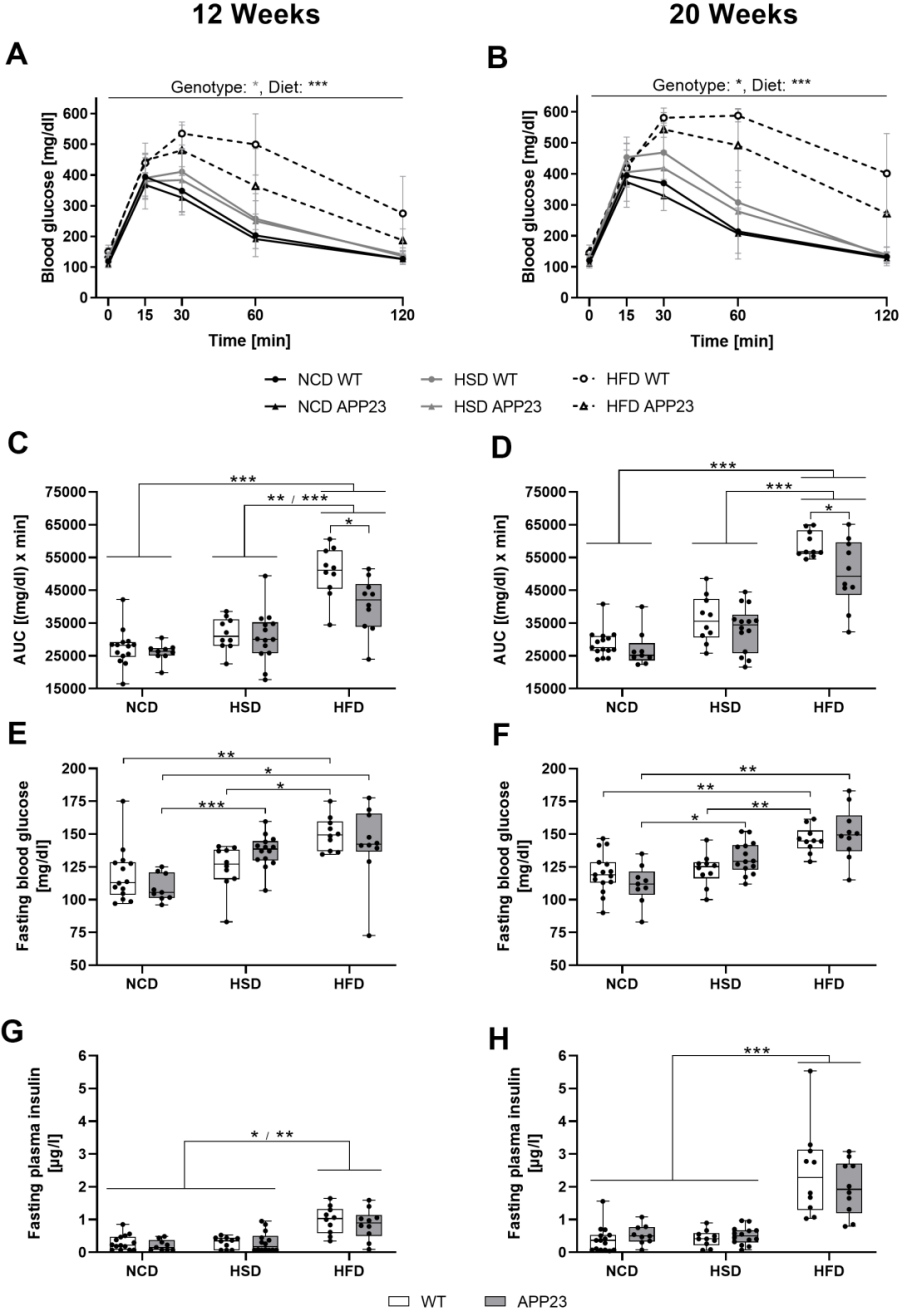
**Acute glucose handling and fasting glucose homeostasis after 12 (left column) and 20 weeks (right column) of dietary interventions in APP23 and WT mice.** (**A**, **B**) Course of blood glucose in an intraperitoneal glucose tolerance test (ipGTT) after 12 (**A**) and 20 weeks (**B**) and the corresponding area under the curve (AUC) (**C**, **D**). The corresponding basal blood glucose (**E**, **F**) and insulin levels (**G**, **H**) of WT and APP23 mice were analyzed after 6 h of fasting in the morning. Data are represented as box (25^th^ to 75^th^ percentile) with median and whiskers from minimum to maximum. Black asterisks indicate significant differences between groups (*: p<0.05; **: p<0.01; ***: p<0.001), gray asterisk indicates a statistical trend towards significance (p<0.1) according to nonparametric ANOVA-type statistics (**A**, **B**) ordinary 2-way ANOVA with Tukey post-hoc test (**C**, **D**) or nonparametric multiple contrast Tukey-type test (**E**–**H**). n_NCD WT_=15, n_NCD APP23_=9, n_HSD WT_=10, n_HSD APP23_=14, n_HFD WT_=10, n_HFD APP23_=10.

To further analyze glucose homeostasis upon glucose challenge, an ipGTT was performed after 12 and 20 weeks (Figure 5, statistics see Supplementary Table 2). After 12 weeks, glucose handling was affected by diet (F(1.941,49.542)=50.159, p<0.001), such as HSD and HFD both delayed the glucose peak (Figure 5A). Additionally, the glucose peak of HFD-fed mice was 30% higher compared to NCD/HSD. Whereas NCD- and HSD-fed mice approximately returned to basal levels after 120 min, glucose levels remained 31% (APP23) and 82% (WT) elevated in HFD-fed mice. APP23 mice showed by trend 9% lower glucose levels than WT mice (F(1.000,49.541)=3.764, p=0.052). This was especially prominent in HFD-fed APP23 mice, which returned to 32% lower final glucose levels than WT mice. After 20 weeks, diet still affected glucose handling (F(1.943,41.614)=82.409, p<0.001) with HSD and HFD delaying the glucose peak, the effect of HFD having even grown (Figure 5B). Furthermore, HFD-fed mice remained far above basal glucose levels after 120 min (WT: 2.7-fold; APP23: 1.8-fold). Still, NCD- and HSD-fed mice nearly returned to fasting glucose levels. Again, APP23 mice showed 9% lower glucose levels compared to WT mice (F(1.000,41.614)=4.183, p=0.041), which was once more especially evident in HFD-fed APP23 mice. Evaluating the area under the curve (AUC) in week 12, HFD-fed mice displayed a significantly larger AUC compared to NCD- and HSD-fed mice (Figure 5C). However, AUC of HFD-fed APP23 mice was 20% smaller compared to HFD-fed WT mice. After 20 weeks, AUC of HFD-fed mice was even more increased compared to NCD- and HSD-fed mice (Figure 5D). Still, HFD-fed APP23 mice displayed a significantly smaller AUC than HFD-fed WT mice, although the difference decreased to 15%.

Fasting glucose levels gradually increased from NCD to HSD to HFD at both time-points (Figure 5E, 5F). HSD elevated fasting glucose levels up to 25% in APP23 but not in WT mice, while HFD increased fasting glucose levels up to 35% in both genotypes. Contrary, fasting insulin levels were only increased in HFD-fed mice (week 12: 3-fold; week 20: 6-fold) in both genotypes (Figure 5G, 5H). Fasting insulin was similar between genotypes. In summary, glucose tolerance was deteriorated by HSD and HFD, with HFD exerting an earlier and stronger effect and APP23 mice being less affected. Moreover, HSD and HFD increased fasting glucose levels, but only HFD elevated fasting insulin levels, both parameters being similar across genotypes.

### APP23 mice showed increased O_2_ consumption, CO_2_ production, energy expenditure, and locomotor activity at night

**Figure 6:**
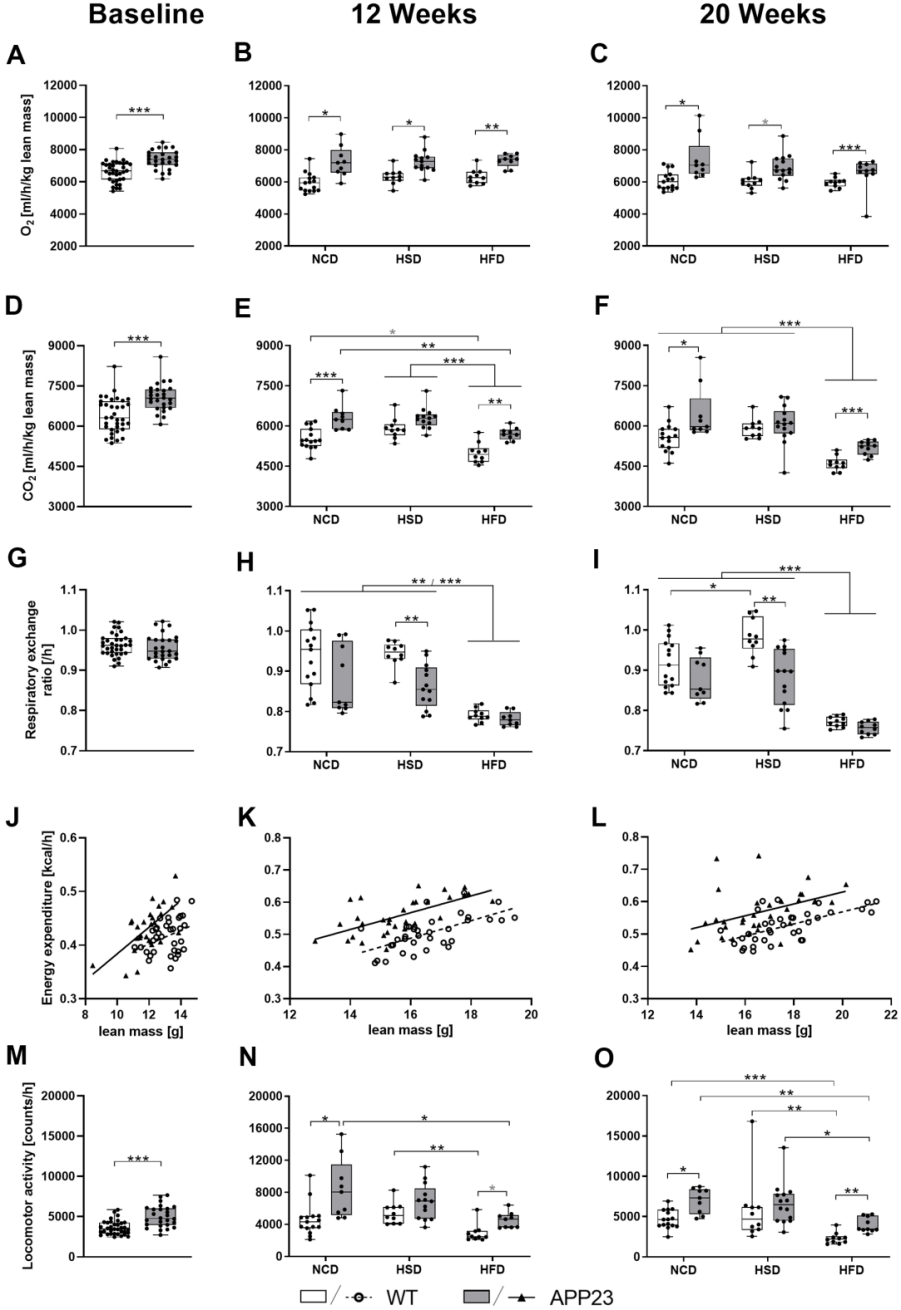
**O_2_ consumption, CO_2_ production, respiratory exchange ratio, energy expenditure, and activity during active phase at baseline (left column), after 12 (middle column) and 20 weeks of diet (right column).** (**A**–**C**) Averaged O_2_ consumption per hour and (**D**–**F**) averaged CO_2_ production per hour (both adjusted for lean mass) as well as (**G**–**I**) corresponding mean respiratory exchange ratio. (**J**–**L**) Averaged energy expenditure per hour correlated with lean mass and (**M**–**O**) averaged locomotor activity per hour. Data are represented as box (25^th^ to 75^th^ percentile) with median and whiskers from minimum to maximum. Black asterisks indicate significant differences between groups (*: p<0.05; **: p<0.01; ***: p<0.001), gray asterisk indicates a statistical trend towards significance (p<0.1) according to nonparametric t-tests (**A**, **D**, **G**, **M**), nonparametric multiple contrast Tukey-type test (**B**, **C**, **E**, **F**, **H**, **I**, **N**, **O**), and Spearman correlation followed by ANCOVA (**J**–**L**). n_NCD WT_=15, n_NCD APP23_=9, n_HSD WT_=10, n_HSD APP23_=14, n_HFD WT_=10, n_HFD APP23_=10.

To further investigate whole-body metabolism of APP23 mice, indirect calorimetry was performed at baseline, week 12 and 20. Figure 6 (statistics see Supplementary Table 3) depicts results measured during active phase. Light phase data are displayed in Supplementary Figure 1. O_2_ consumption and CO_2_ production adjusted for lean mass were increased in APP23 mice at any time-point. Measurements adjusted for body weight were similar (Supplementary Figure 2). On closer examination, APP23 mice consumed 12% more O_2_ at baseline (Figure 6A) and up to 22% in week 12 and 20 (Figure 6B, 6C). Diet alone did not affect O_2_ consumption at any time-point. Correspondingly, CO_2_ production was 10% elevated in APP23 mice at baseline (Figure 6D) and up to 14% in week 12 and 20 (Figure 6E, 6F). Contrary to O_2_ consumption, CO_2_ production was affected by diet, such as HFD-fed mice produced up to 22% less CO_2_ compared to NCD/HSD at both time-points. The respiratory exchange ratio was similar between genotypes at baseline (Figure 6G). During HSD, it decreased up to 10% in APP23 mice at both time-points (Figure 6H, 6I), while no genotype differences were observed during NCD and HFD. HSD increased the respiratory exchange ratio, however only in WT mice and week 20, whereas HFD decreased it up to 16% in both genotypes at both time-points.

Next, we evaluated energy expenditure and activity. To account for the major contribution of body weight, energy expenditure was correlated with lean mass (Figure 6J–6L and Supplementary Figure 1J–1L) or body weight (Supplementary Figure 2G–2I, 2P–2R; correlation statistics see Supplementary Table 4). Since diet alone did not affect energy expenditure, different dietary groups were pooled. APP23 mice showed 11% elevated energy expenditure compared to WT mice at baseline (F(1,58)=15.488, p<0.001 lean mass-corrected; Figure 6J), 9% in week 12 (F(1,63)=51.102, p<0.001 lean mass-corrected; Figure 6K) and 10% in week 20 (F(1,65)=21.948, p<0.001 lean mass-corrected; Figure 6L). Supplementary Figure 3 shows energy expenditure over the course of 24 h revealing a constantly increased energy expenditure of APP23 mice, especially pronounced during dark phase. Correspondingly, APP23 mice were 38% more active than WT mice at baseline (Figure 6M) and up to 84% and 71% in week 12 and 20 (Figure 6N, 6O). HFD feeding decreased locomotor activity up to 60% compared to NCD/HSD in both genotypes at both time-points.

Altogether, APP23 mice showed notably higher O_2_ consumption, CO_2_ production, and energy expenditure as well as increased activity. The effects of diet were mild and mainly represented by an HFD-induced reduction of activity and – as expected – of the respiratory exchange ratio.

### Proteome analyses indicated potential mitochondrial dysfunction of APP23 mice

**Figure 7:**
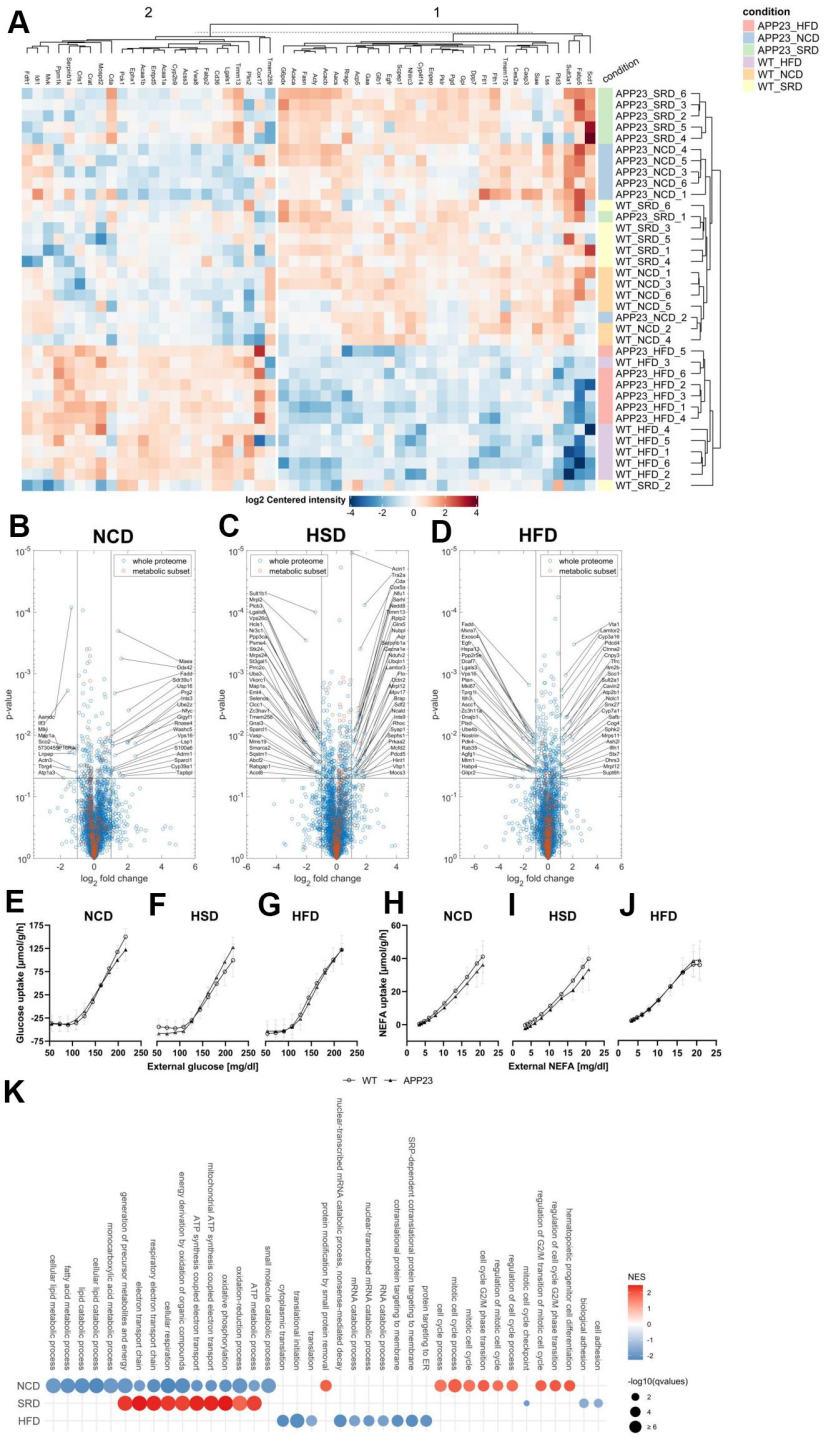
**Proteome analyses of liver tissue.** (**A**) Heatmap of differentially regulated proteins (**B**–**D**) Volcano plots of APP23 versus WT mice within each diet. (**E**–**G**) Simulated hepatic glucose tolerance of APP23 versus WT mice within each diet. (**H**–**J**) Simulated hepatic NEFA tolerance of APP23 versus WT mice within each diet. (**K**) GSEA of APP23 versus WT mice within each diet. (**B**, **E**, **H**) NCD; (**C**, **F**, **I**) HSD; (**D**, **G**, **J**) HFD. n=6 each.

To investigate metabolic alterations in APP23 mice on protein level, we performed proteome analyses in liver (Figure 7) and brain (Supplementary Figure 4). Liver proteome profiles showed a genotype-clustering within NCD- and HSD-fed mice but a genotype-overlapping clustering within HFD-fed mice. (Figure 7A). Volcano plots displayed several differentially regulated proteins in APP23 mice, especially involved in lipid metabolism (CYP39A1, SDR39U1, AAMDC, FTO, ACOT8, VASP), oxidative phosphorylation, β-oxidation and general mitochondrial function (TBRG4, SCO2, MPV17, MRPL12, NDUFV2, NUBBPL, GLRX5, TIMM13, NFU1, COX5A, SCO1, CYP3A16, PDK4) or related to inflammation and metabolic stress (PRKAA2, SERPINB1A, TMEM258, LGALS3; Figure 7B–7D). Some mitochondria-related proteins were also differentially regulated in brain proteome of APP23 mice (MCEE, ADCK1) but the majority is involved in signal transduction, vesicle trafficking, synaptic function and neuronal plasticity (SYT17, DIP2A, BLOC1S1, SNCB, BAIAP3, WDR11, KCNIP3, TRAPPC13; Supplementary Figure 4). Simulation of metabolic capacities showed only minor differences in hepatic glucose tolerance. NCD-fed APP23 mice had slightly better glucose tolerance at low external glucose, while their glucose tolerance was slightly inferior at high external glucose (F(9,90)=2.710, p=0.008), contrary during HSD (F(9,90)=5.750, p<0.001) and without difference during HFD (Figure 7E–7G). NEFA tolerance seemed to be slightly decreased in NCD- and HSD-fed APP23 mice (n.s.; Figure 7H–7J). Gene Set Enrichment Analysis (GSEA) revealed downregulation of protein sets affecting lipid metabolism, oxidative phosphorylation, and mitochondrial function in NCD-fed APP23 mice (Figure 7K). Interestingly, the same protein sets were upregulated in HSD-fed APP23 mice but unchanged in HFD-fed APP23 mice. Instead, protein sets contributing to translation and amino acid metabolism were downregulated.

## DISCUSSION

Involuntary weight loss advances with AD progression resulting in poorer health, reduced quality of life, and increased mortality [14]. Thus, identifying the role of (reduced) body weight in AD might contribute to the understanding of metabolic implications in AD pathogenesis. Therefore, we investigated APP23 mice during diet-induced metabolic challenge. To our knowledge, this is the first longitudinal study assessing metabolic features of APP23 females, revealing lower body weight, less adipocyte hypertrophy as well as steatosis, elevated energy expenditure as well as activity and evidence for potential mitochondrial damage.

A lower body weight already emerges in 4-week old APP23 mice due to lower lean mass, whereas plaque development starts around 6 months of age [20]. However, lower body weight of APP23 mice did not result from smaller body length, which was also reported for 24-months old APP23 females [21]. Although APP23 mice do not model AD-related weight loss, their lower body weight at a pre-plaque stage corresponds to lower body weight of AD patients, which arises from early weight loss prior to the onset of clinical AD symptoms [16, 22]. Consistent with literature, HSD induced a mild, but HFD a substantial body weight gain [19, 23–25], in which APP23 mice gained less fat mass. Analysis of food intake as a potential mechanism for lower body weight revealed lower HFD consumption, contradicting a study in APP23 males reporting increased food intake [26]. However, estrogen might have influenced food intake via its interaction with leptin [27], counterbalancing food intake in APP23 females. HFD contains less carbohydrates than NCD and HSD but similar amounts of protein, which may explain the increased HFD-intake of WT mice, as mice adapt their food intake to a favored low protein/high carbohydrate ratio even by overeating [28]. In contrast, food intake of APP23 mice remained constant regardless of diet suggesting potential alterations of nutrient-specific feedback mechanisms. This is corroborated by a study in Tg2576 mice (same *App* mutation/different promoter) revealing dysfunctional hypothalamic leptin signaling depicted by decreased leptin levels [29]. The fact that hyperactivity and increased energy expenditure of APP23 mice were not compensated by higher food intake, as one could have expected [30], also suggests a dysregulation of energy balance. Since the hypothalamus plays a crucial role in regulating food intake and energy balance [31], altered nutrient-specific feedback and lacking compensatory hyperphagia in APP23 mice might be caused by altered hypothalamic leptin signaling [32]. Altogether, although APP23 mice displayed only reduced food intake during HFD, compensatory hyperphagia to account for increased energy expenditure was not observed during any diet and, thus, potentially contributing to lower body weight of APP23 mice. As a potential underlying mechanism, hypothalamic leptin signaling in APP23 mice deserves further investigations.

Necropsy showed that while HSD induced mild liver weight gain only in WT mice, HFD induced substantial liver weight gain in both genotypes. Histology and triglyceride quantification revealed progressive steatosis, however diminished in APP23 mice, especially during HFD. The lower degree of steatosis in APP23 mice corresponds to their lower body weight. Indeed, it has been shown in weight loss experiments that body weight strongly influences the extent of hepatic steatosis [33]. Furthermore, steatosis considerably contributes to insulin resistance via hepatic inflammation [34]. Actually, the degree of steatosis was to some extent reflected in insulin levels: HFD-fed WT mice showed the most prominent steatosis accompanied by the most prominent elevation of insulin levels, while the lower extent of steatosis in HFD-fed APP23 mice might contribute to a less prominent insulin resistance. HSD related body weight gain and the corresponding degree of steatosis might not be sufficient to induce insulin resistance. Consistently, only HFD but not HSD elevated eWAT weight. Adipocyte size gradually increased from NCD to HFD, APP23 mice exhibiting less hypertrophy. Hypertrophic instead of hyperplastic adipose tissue expansion indicates metabolic disease [35]. Thus, HSD-induced hypertrophy without increased fat mass suggests an incipient metabolic dysfunction.

We longitudinally monitored glycemic control via fed insulin concentrations. Gradually increasing insulin levels during HFD indicate a compensation of beginning insulin resistance via higher insulin secretion to maintain physiological glucose levels. As insulin sensitivity is strongly correlated with body weight [36], lower body weight of APP23 mice together with the lesser degree of steatosis potentially accounts for lower insulin levels in HFD-fed APP23 mice suggesting superior insulin sensitivity. Furthermore, we examined corticosterone as a functional antagonist of insulin and NEFA as a correlate for lipolysis. Corticosterone levels were similar between genotypes as already described [19], possibly due to young age and low Aβ load of APP23 mice [37]. NEFA levels were unaltered by genotype but reduced by HFD, potentially due to the lipolysis-inhibiting effect of elevated insulin levels [38]. This observation rather points to a still physiological insulin sensitivity, as obesity and insulin resistance lead to increased lipolysis [39]. Additionally, fasting glucose and insulin levels were unaltered in APP23 mice.

Although reduced brain glucose metabolism occurs early in AD progression [40], peripheral glycemic dysfunction is under debate and was not observed in APP23 mice. Evidence suggests that glycemic dysregulation in AD might be mediated by peripheral Aβ [41], which is low in APP23 mice [42]. Together with lower body weight and less steatosis this might account for an even slightly superior peripheral metabolism supporting current literature [43]. HFD considerably deteriorated glucose tolerance already after 12 weeks potentially via a combination of elevated hepatic and adipose tissue inflammation, lipid overload and hypothalamic alterations [44]. HSD aggravated glucose tolerance only moderately after 20 weeks, possibly due to the scarce effect on body weight considering obesity a major contributor to glucose intolerance.

To further investigate mechanisms underlying lower body weight of APP23 mice, indirect calorimetry was evaluated. We showed for the first time that APP23 mice consumed notably more O_2_ and produced notably more CO_2_, indicating an increased metabolic rate even before Aβ plaque development. This finding confirms a study in pre-plaque Tg2576 mice with increased O_2_ consumption also at rest [29]. Interestingly, the respiratory exchange ratio of NCD- and HSD-fed APP23 mice was decreased, suggesting a tendency towards fat instead of carbohydrates burning, potentially contributing to lower body weight and adiposity in APP23 mice. Furthermore, energy expenditure was elevated in APP23 mice, accompanied by hyperactivity. Hyperactivity has previously been observed in APP23 males, especially towards the end of the active phase, potentially reflecting so-called sundowning behavior of AD patients [45]. Whereas energy expenditure was unaffected by diet, activity was reduced during HFD as described [46]. The finding that HFD reduced activity but not energy expenditure and O_2_ consumption, suggests that elevated energy expenditure of APP23 mice might not solely depend on increased activity. Altogether, this indicates that increased O_2_ consumption, CO_2_ production, and energy expenditure of APP23 mice are probably not only caused by hyperactivity but additionally by an increased resting metabolic rate.

Interestingly, our proteome analyses imply a mitochondrial dysfunction of APP23 mice. Studies indicate that dysfunctions of cerebral mitochondria, potentially induced by Aβ, might play a central role in AD pathogenesis [47]. Earlier proteome studies in APP23 brains revealed altered expression of proteins associated with glycolysis and oxidative phosphorylation as well as increased oxidative stress represented by a higher proportion of carbonylated proteins [48]. We also report alterations in mitochondria-related proteins in brain tissue. Even more interesting, we observed very similar modifications in liver tissue, revealing differential expression of proteins involved in oxidative phosphorylation, β-oxidation, and metabolic stress. Hence, our data might indicate a general mitochondrial dysfunction in APP23 mice not only limited to neurons. Notably, this dysfunction seems to be counterbalanced by HSD, potentially due to massive substrate provision (i.e. sucrose) possibly stimulating mitochondrial pathways. It has been shown previously that HSD induces overexpression of specific mitochondrial proteins in hepatocytes and increases the mitochondrial production of reactive oxygen species [49]. This reaction – normally a sign of oxidative stress – might be beneficial in HSD-fed APP23 mice to compensate for mitochondrial dysfunction represented by downregulated mitochondrial proteins in NCD-fed APP23 mice. This is in line with slightly inferior hepatic glucose tolerance in NCD-fed APP23 mice compared to slightly superior hepatic glucose tolerance in HSD-fed APP23 mice. As HFD and hepatic steatosis have been shown to induce oxidative stress and mitochondrial damage [50], we assumed a differential regulation of mitochondrial pathways in HFD-fed mice, however, found no such evidence. Moreover, a transcriptome analysis of HFD-fed APP23 brains has shown higher expression of genes involved in immune response and inflammation due to HFD [25], which was not obvious in our brain tissue on protein level. However, mice in the mentioned study were 12-months old, thus potentially being more prone to inflammation due to their advanced plaque pathology.

In summary, this study is the first to systematically analyze lower body weight of pre-plaque APP23 mice, extensively characterizing metabolic features of this murine AD model. Although our data is descriptive, we conclude that lower body weight of APP23 mice is most likely caused by a combination of hyperactivity, increased metabolism, and dysregulated energy balance reflected by the absence of compensatory feeding mechanisms. Furthermore, we found evidence of dysfunctional mitochondrial pathways not only in the brain but for the first time also in the liver. These findings are highly interesting with regard to emerging hypotheses about the implication of mitochondrial dysfunction in AD pathogenesis. Both, mitochondrial dysfunction as well as altered hypothalamic leptin signaling deserve further research as they might underlie the described metabolic changes in APP23 females. Interestingly, lower body weight of APP23 mice may partially protect from diet-induced metabolic stress depicted by extenuated steatosis and adipocyte hypertrophy, which seem to contribute to improved insulin sensitivity. As the translation of basic animal research into the clinic is challenging, we strongly encourage thorough metabolic investigation of AD patients to contribute to the understanding of metabolic implications in AD pathogenesis.

## MATERIALS AND METHODS

### Ethics statement

This study was approved by the local animal ethics committee (LAGeSo Berlin; G0074/16) and carried out in accordance with EU Directive 10/63/EU as well as in line with the ARRIVE guidelines.

### Animal experiments

The study was conducted in 33 female transgenic APP23 mice (APP23) and 35 female healthy littermates (WT) on a C57BL/6J background. APP23 mice overexpress human APP_751_ cDNA with the Swedish double mutation under the murine Thy-1 promotor [20]. Aβ plaque deposition starts at 6 months of age [20]. Mice were group-housed (2-3/cage) in environmentally controlled, individually ventilated cages (21.5° C±1.5) with a 12-hour light/dark cycle and ad libitum access to food and water. 4-6-week old mice were randomly allocated to normal-control (NCD), high-sucrose (HSD) or high-fat diet (HFD; all Research Diets) for 20 weeks (Figure 1B and Supplementary Table 1). Body weight and food intake were recorded weekly (Figure 1A). Body length was exemplarily measured from tip of the nose to tail root in a separate set of young adult mice (8-11-week old female and male APP23 (n=11, thereof 4 females) and WT mice (n=8, thereof 2 females)). Monthly, body composition was measured by ^1^H-magnetic-resonance-spectroscopy (NMR) using a Minispec LF50 (Bruker) and blood was collected from *Vena facialis* in the morning. Serum was stored at -80° C for analysis of insulin levels. Indirect calorimetry and activity were analyzed in single-caged mice (LabMaster-System, TSE) for 48 h (12 h adaption/36 h evaluation: 1 light and mean of 2 dark phases) in week 0, 12, and 20. Intraperitoneal (i.p.) glucose tolerance tests (ipGTT) were performed in week 12 and 20 after a 6 h morning-fast and local analgesia of tail tip (lidocaine/prilocain, AstraZeneca). Glucose levels were measured in duplicates (Contour Next, Bayer) before (0 min), 15, 30, 60, and 120 min after injection of 2 g/kg body weight glucose. At 0 min, plasma was additionally collected and stored at -80° C for analysis of fasting insulin levels. Terminally, mice were sacrificed as described [19]. Plasma was stored at -80° C. Tissues were stored partially snap-frozen (-80° C) and partially in 4% PFA/PBS (4° C).

### Histology and immunohistochemistry

PFA-fixed livers were transferred in O.C.T. Compound (Sakura) and 5 μm sections were cryocut using a Jung Frigocut 2800E (Leica). eWAT was dehydrated, infiltrated and embedded with paraffin and 5 μm sections were cut using a Microm Cool-Cut HM 325 (ThermoFisher).

eWAT and liver sections were stained with hematoxylin/eosin and Oil Red O, respectively, as described [51]. Hematoxylin/eosin-stained eWAT (4 sections of 6 random mice/genotype/diet) and Oil Red O-stained liver (4 sections of 8 random mice/genotype/diet) were recorded with 20x on a BZ-9000 microscope (Keyence). Average adipocyte size and mean percentage Oil Red O-covered area were analyzed with ImageJ (V1.52a).

### Biochemical analyses

Triglycerides were quantified in liver tissue (6 random mice/genotype/diet). Frozen liver (~60 mg) was suspended in 600 μl alcoholic KOH and incubated at 60° C for 6 h. 540 μl 1M MgCl_2_ were added to 500 μl of the saponificated sample, followed by 10 min incubation on ice and 30 min centrifugation at full speed. Supernatant was collected and analyzed according to manufacturer instructions using the Triglycerides FS 10’ kit (Diagnostic Systems). Triglyceride concentrations were multiplied by actual liver weight measured upon sacrifice to obtain absolute values.

Fed insulin was quantified in serum and fasting insulin in plasma using the Mouse Insulin ELISA kit (Mercodia). Corticosterone and NEFAs were measured in final plasma using the Corticosterone ELISA kit (IBL International) and the NEFA-HR(2) kit (Wako).

### Proteomics

Frozen whole-brain (350-450 mg, 4 random mice/genotype/diet) was prepared and homogenized. 18-55 mg were used for lysates, 30 μg digested and 1 μg was analyzed using nano-LC-MS/MS as described [52, 53]. Data were processed with MaxQuant (V1.6.0.1) [54] and searched against mouse UniProtKB with 21,074 entries, released 12/2018.

Frozen liver (~5mg, 6 random mice/genotype/diet) was suspended in 75 μl lysis buffer (1% SDS/100 mM ABC+1.25x PIC), sonicated twice (Covaris ML230: PIP 375 W, DF 25%, CPB 50, 20 repeats, 10 s pulse, 10 s delay, 12 C, dither 3 mm Y-axis) and insoluble particles were removed by centrifugation. 15 μl lysate was adjusted to 50 μl with water and processed on a Biomek i7 workstation using protocol SP3 [55]. On a two-column-system 1.25 μg tryptic peptides were analyzed by LC-MS/MS [53]. Separation was done by applying a gradient from 7.5% to 55% B in 120 min at a flow rate of 300 nL/min (solvent A: 0.1% formic acid in water; solvent B: 80% acetonitrile and 0.1% formic acid). The Orbitrap was configured to acquire 25 × 24 m/z (covering 400-1000 m/z), precursor isolation window data independent acquisition (DIA) spectra (17,500 resolution, AGC target 1e6, maximum inject time 60ms, normalized HCD collision energy 27%) using overlapping windows. A full scan MS spectra (m/z 400-1000) was recorded at 35,000 resolution after 60 ms accumulation of ions to a 1e6 target value in profile mode. Raw data were processed using DIA-NN 1.7.12 [56]*,* scan window size set to 14 and MS2 and MS1 mass accuracies to 20 and 10 ppm, respectively. A project-independent public spectral library [57] and mouse UniProt (UP000000589) were used for annotation. The library was automatically refined based on the dataset, global q=0.01 (using the “Generate spectral library” option in DIA-NN) [58].

### Modeling of metabolic capacities

Metabolic capabilities of individual mice were evaluated using an established kinetic model of the energy metabolism of hepatic and neuronal cells encompassing glycolysis, citric acid cycle, the respiratory chain and oxidative phosphorylation [59, 60]. This model describes the dynamic of metabolites and fluxes via ordinary differential equations taking into account the regulatory properties of the underlying metabolic enzymes, such as substrate affinities (K_m_-values), allosteric properties (K_i_-values and K_a_-values) and alterations in these parameters due to phosphorylation (interconversion). It distinguished between different cellular compartments (cytosol, mitochondria, endoplasmatic reticulum). As maximal enzyme activity is proportional to protein abundance, individualized models were generated scaling the maximal enzyme activities (V_max_) of each metabolic enzyme and transporter by the relation $V_{max}^{animal}=V_{max}^{control}\cdot \frac{E^{animal}}{E^{control}},$ where *E^control^* is the average enzyme intensity in all controls and *E^animal^* is the enzyme concentration in the individual animal. Metabolic capacities were simulated by systemic variation of the external conditions (plasma nutrient and hormone composition). Hepatic glucose exchange flux was simulated by simultaneous variation of plasma glucose concentration, plasma free fatty acid concentration and plasma insulin and glucagon concentrations as in [59, 61, 62]. MATLAB (Release2012a; optimization toolbox) was used for simulations and respective graphs.

### Statistics

Data are represented as boxes (25^th^ to 75^th^ percentile) with median and min/max-whiskers or as mean with standard deviation. SPSS Statistics (V25), GraphPad Prism (V8), and R (V3.6.3; packages nparcomp, nparLD, car, DEP, clusterProfiler) were used for statistics and graphs.

MS output was filtered (FDR=0.01) at peptide level and for completeness in at least one experimental group, missing values were imputed and all contrasts between experimental groups were analyzed for differentially expressed proteins (α≤0.05, absolute log2 fold-change≥1). Volcano plots and heatmaps were automatically generated. For GSEA, data were mapped to the human annotation and biological processes from the gene ontology database were selected. Results were filtered (20-500 gene set size, q≤0.01) and the top-10 enriched terms per contrast and direction were plotted.

Shapiro-Wilk test for normal distribution and Levene’s test for equality of variances were applied. If passed, 2-way ANOVA (ordinary/repeated measures) with Tukey post-hoc test was performed. Otherwise, nonparametric t-test, nonparametric multiple contrast test type Tukey, or nonparametric (repeated measures) ANOVA-type statistics were chosen. According to [63], ANCOVA was performed for analysis of pooled energy expenditure data to correct for lean mass-/body weight-effects. Therefore, equality of slopes was checked by simple linear regression before pooling dietary groups in a first step, energy expenditure was correlated with lean mass/body weight using Spearman correlation in a second step and finally ANCOVA was performed. Significance was considered *p≤0.05, **p≤0.01, ***p≤0.001, trends towards significance (p<0.1) are displayed in gray. No data were excluded from any analysis.

### Data availability

The datasets generated and/or analyzed during the current study are available from the corresponding author on reasonable request.

## Supplementary Material

Supplementary Figures

Supplementary Tables
