# Supplementary material for: Dietary-challenged mice with Alzheimer-like pathology show increased energy expenditure and reduced adipocyte hypertrophy and steatosis
Source: Aging (Albany NY). 2021 Apr 16;13(8):10891–919. doi: 10.18632/aging.202978 (PMC8109068; doi:10.18632/aging.202978)
Supplement: Supplementary Figures [file aging-13-202978-s001.pdf]

SUPPLEMENTARY FIGURES

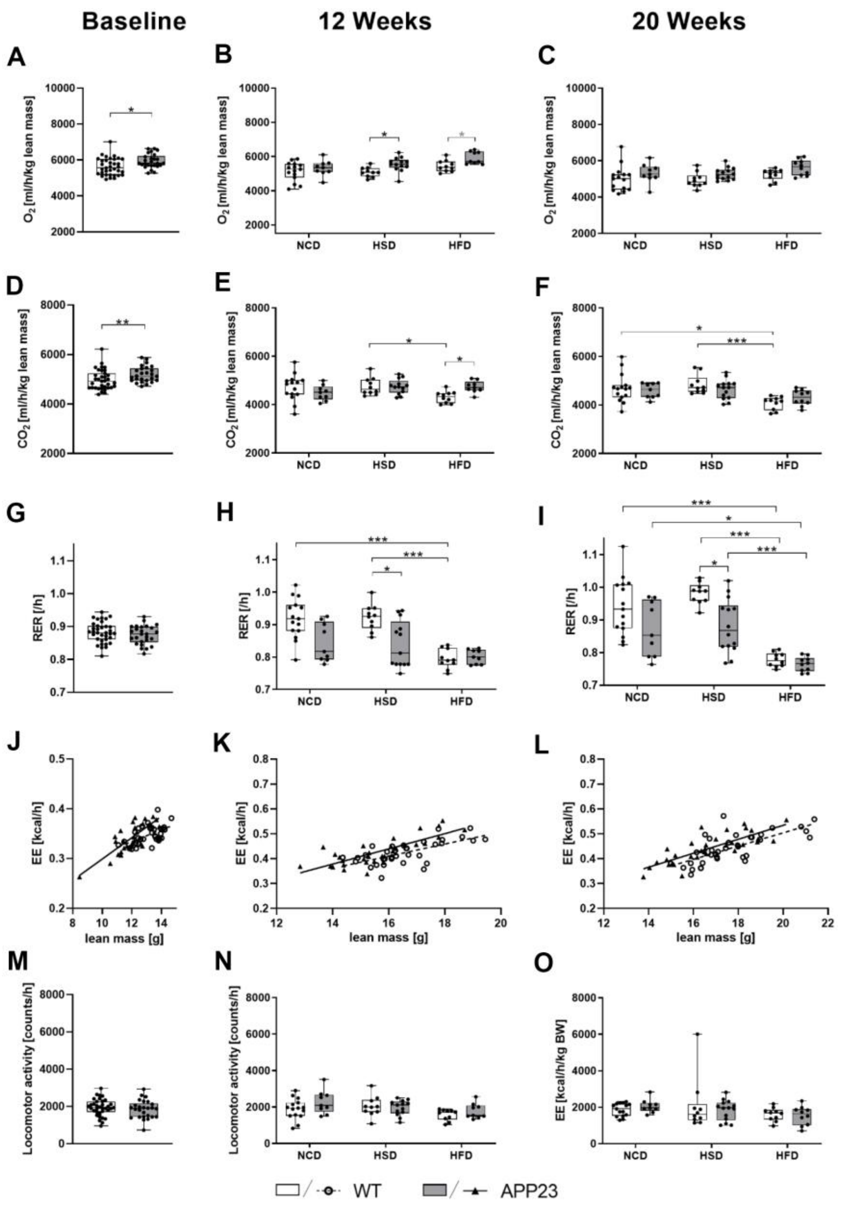

Supplementary Figure 1. Oxygen (O_2) consumption, carbon dioxide (CO_2) production, respiratory exchange ratio (RER), energy expenditure (EE), and locomotor activity of mice measured in metabolic cages during the light (inactive) phase at baseline (left column) and after 12 (middle column) and 20 weeks of diet (right column). (**A**–**C**) Averaged O_2 consumption per hour and (**D**–**F**) averaged CO_2 production per hour (both adjusted for lean mass) as well as (**G**–**I**) corresponding mean RER at baseline (**A**, **D**, **G**), after 12 (**B**, **E**, **H**) and 20 weeks of intervention (**C**, **F**, **I**). (**J**–**L**) Averaged EE per hour correlated with lean mass and (**M**–**O**) averaged locomotor activity per hour at baseline (**J**, **M**) and after 12 (**K**, **N**) and 20 weeks of diet (**L**, **O**). Metabolic cage data was collected after 12 h adaption time during 36 h analysis (1 light and mean of 2 dark phases) with 2 cycles per hour. Date of light and dark phase were analyzed separately. Data are shown averaged per hour and except for RER and locomotor activity adjusted for lean mass. Data are represented as box (25th to 75th percentile) with median and whiskers from minimum to maximum. Black asterisks indicate significant differences between groups (*: $p < 0.05$; **: $p < 0.01$; ***: $p < 0.001$), gray asterisk indicates a statistical trend towards significance ($p<0.1$) according to nonparametric t-tests (**A**, **D**, **G**, **J**, **M**) or nonparametric multiple contrast Tukey-type test (**B**, **C**, **E**, **F**, **H**, **I**, **K**, **L**, **N**, **O**). Abbreviations: WT: wild type control; APP23: transgenic mouse model; NCD: normal-control diet; HSD: high-sucrose diet; HFD: high-fat diet; RER: respiratory exchange ratio; EE: energy expenditure.

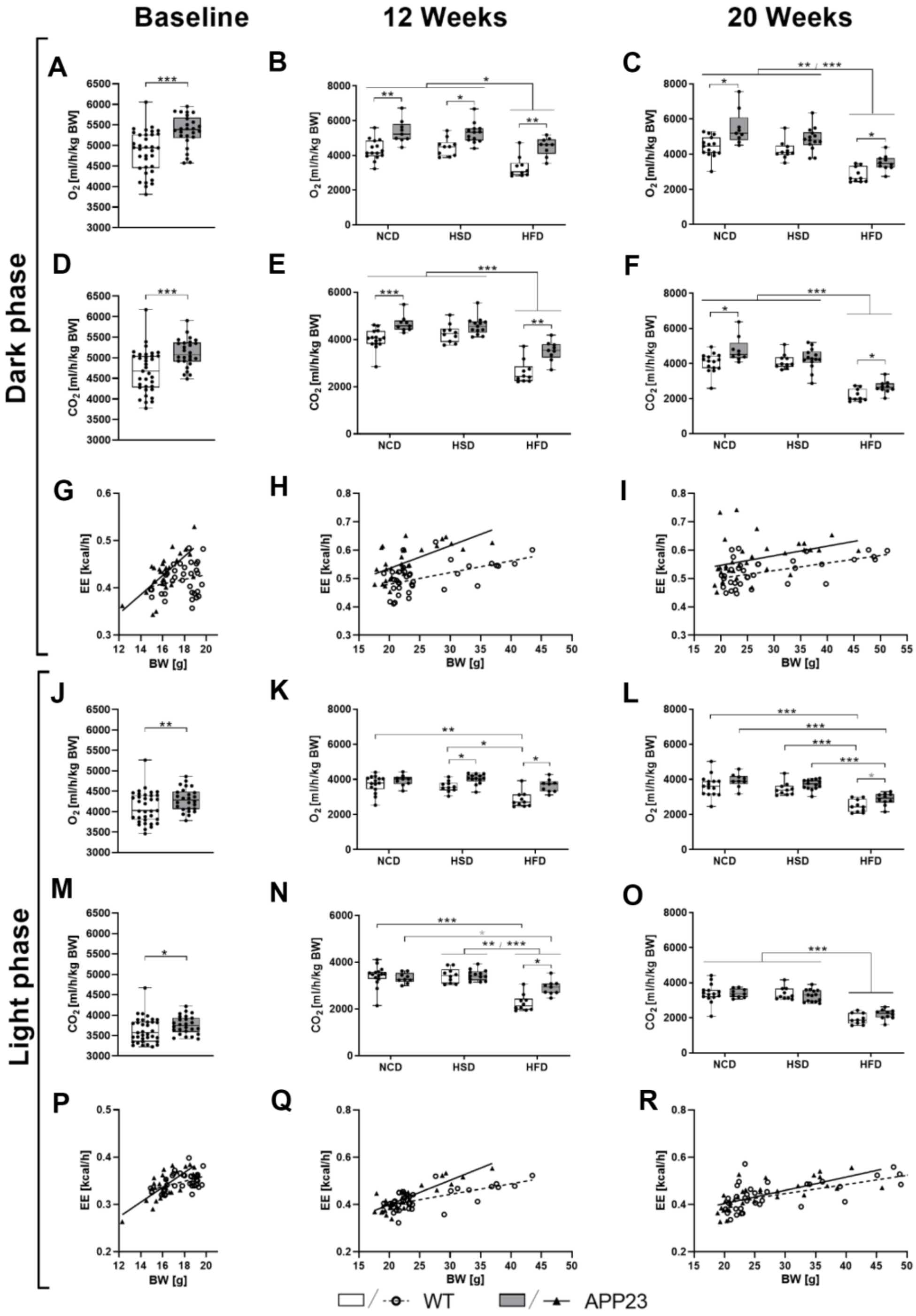

Supplementary Figure 2. Oxygen (O_2) consumption, carbon dioxide (CO_2) production, and, energy expenditure (EE) of mice measured in metabolic cages during the light (inactive) and dark (active) phase at baseline (left column) and after 12 (middle column) and 20 weeks of diet (right column). (**A**–**C**) and (**J**–**L**) Averaged O_2 consumption per hour adjusted for total BW during dark (active) phase (**A**–**C**) and during light (inactive) phase (**J**–**L**). (**D**–**F**) and (**M**–**O**) Averaged CO_2 production per hour adjusted for total BW during dark (active) phase (**D**–**F**) and during light (inactive) phase (**M**–**O**). (**G**–**I**) and (**P**–**R**) Averaged EE per hour correlated with total BW during dark (active) phase (**G**–**I**) and during light (inactive) phase (**P**–**R**). Metabolic cage data was collected after 12 h adaption time during 36 h analysis (1 light and mean of 2 dark phases) with 2 cycles per hour. Date of light and dark phase were analyzed separately. Data are shown averaged per hour and except for RER and locomotor activity adjusted for lean mass. Data are represented as box (25th to 75th percentile) with median and whiskers from minimum to maximum. Black asterisks indicate significant differences between groups (*: $p < 0.05$; **: $p < 0.01$; ***: $p < 0.001$), gray asterisk indicates a statistical trend towards significance ($p<0.1$) according to nonparametric t-tests (**A**, **D**, **G**, **J**, **M**) or nonparametric multiple contrast Tukey-type test (**B**, **C**, **E**, **F**, **H**, **I**, **K**, **L**, **N**, **O**). Abbreviations: WT: wild type control; APP23: transgenic mouse model; NCD: normal-control diet; HSD: high-sucrose diet; HFD: high-fat diet; EE: energy expenditure.

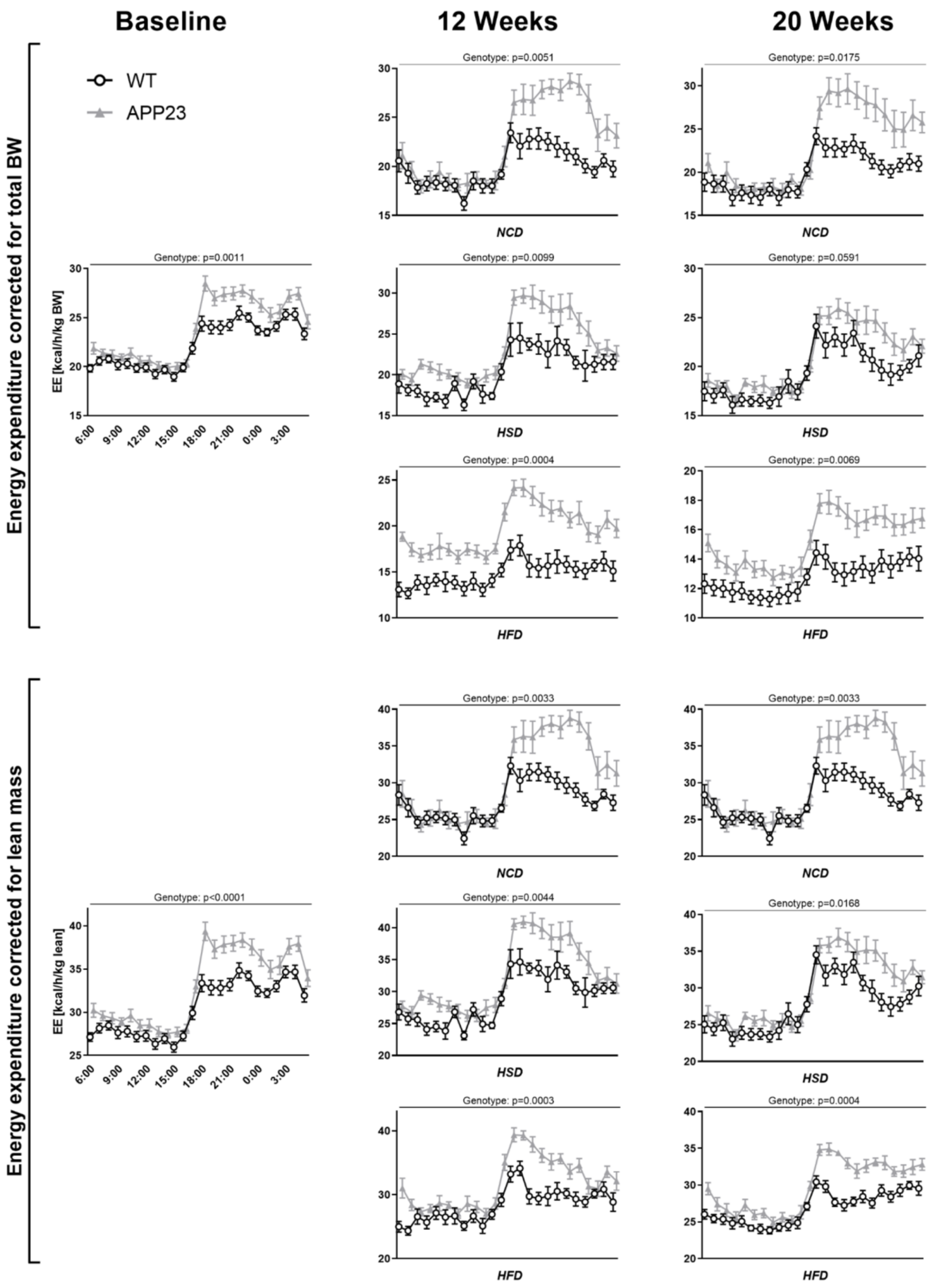

Supplementary Figure 3. Energy expenditure (EE) over the course of 24 hours (one light, one dark phase) at baseline (left column) and after 12 (middle column) and 20 weeks of diet (right column). Upper panel: Energy expenditure corrected for total body weight (BW). Lower panel: Energy expenditure corrected for lean mass. APP23 mice showed increased EE compared to WT regardless of time-point and diet. Data are represented as mean with standard deviation. Repeated measures 2-way ANOVA was performed. Abbreviations: WT: wild type control; APP23: transgenic mouse model; NCD: normal-control diet; HSD: high-sucrose diet; HFD: high-fat diet; BW: body weight; EE: energy expenditure.

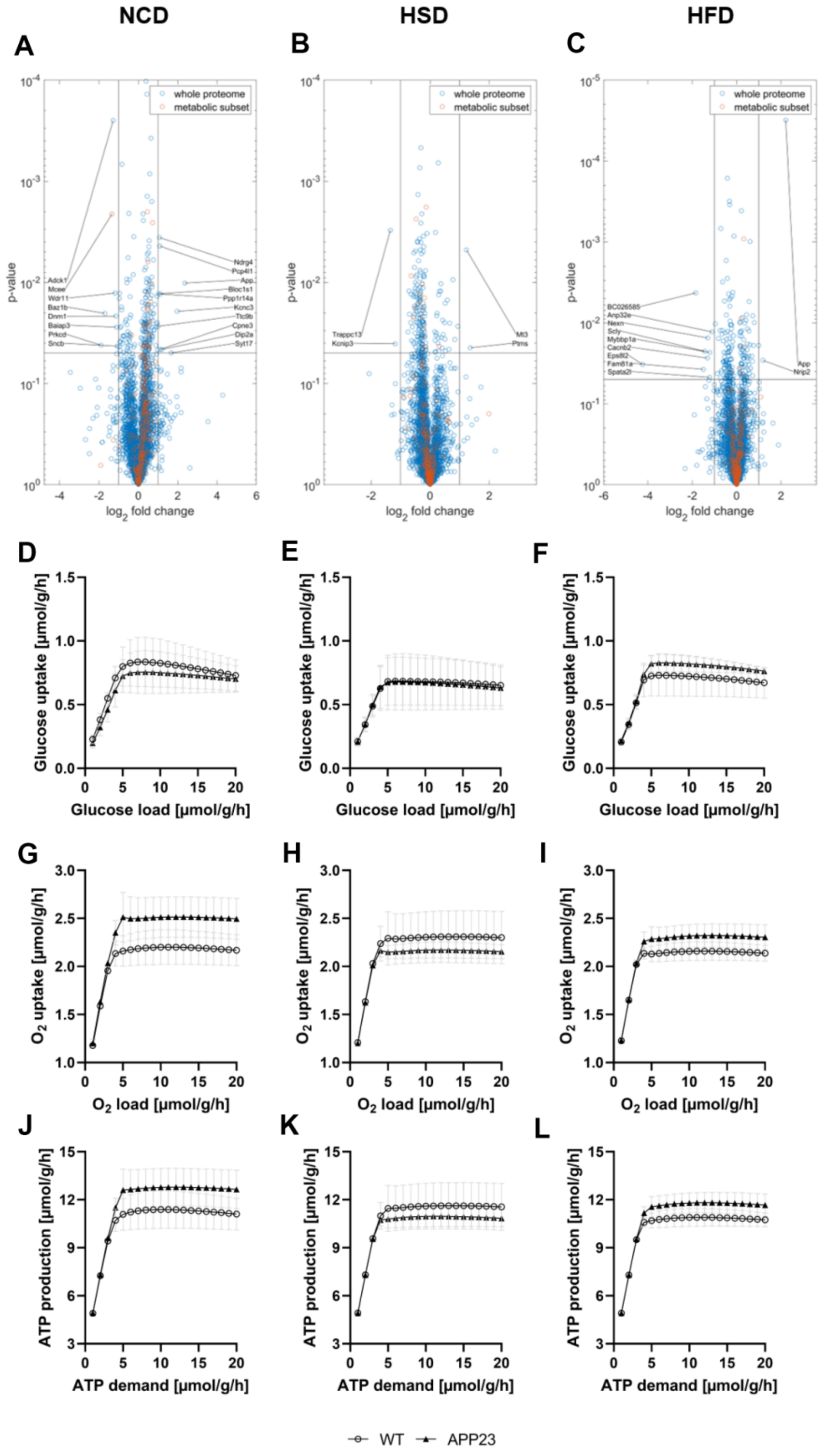

Supplementary Figure 4. Proteome analysis of brain tissue. (**A**–**C**) Volcano plots of APP23 mice versus WT mice within each diet. Such as seen in liver proteome profiles, some mitochondria-related genes were differentially regulated in brain proteome profiles of APP23 mice (MCEE, ADCK1). However, the majority of differentially regulated proteins is involved in signal transduction, vesicle trafficking, synaptic

function and neuronal plasticity (Syt17, DIP2a, BLOC1s1, SNCB, BAIAP3, WDR11, KCNIP3, TRAPPC13). (**D**–**F**) Simulated neuronal glucose tolerance of APP23 mice versus WT mice within each diet. Simulated neuronal glucose tolerance did not differ between genotypes and dietary groups. (**G**–**I**) Simulated neuronal oxygen consumption of APP23 mice versus WT mice within each diet. (**J**–**L**) Simulated neuronal ATP production of APP23 mice versus WT mice within each diet. Simulated neuronal oxygen consumption and ATP production were slightly elevated in NCD-fed APP23 mice, which might be an overcompensation of beginning mitochondrial dysfunction, resulting in yet unaltered glucose tolerance. Thus, similar analyses in aged APP23 mice with advanced AD pathology would be of interest. Differences in HSD- and HFD-fed APP23 mice were too small to be interpreted. (**A**, **D**, **G**, **J**) NCD; (**B**, **E**, **H**, **K**) HSD; (**C**, **F**, **I**, **L**) HFD. Abbreviations: WT: wild type control; APP23: transgenic mouse model; NCD: normal-control diet; HSD: high-sucrose diet; HFD: high-fat diet.
