# Supplementary material for: Dietary-challenged mice with Alzheimer-like pathology show increased energy expenditure and reduced adipocyte hypertrophy and steatosis
Source: Aging (Albany NY). 2021 Apr 16;13(8):10891–919. doi: 10.18632/aging.202978 (PMC8109068; doi:10.18632/aging.202978)
Supplement: Supplementary Tables [file aging-13-202978-s002.pdf]

SUPPLEMENTARY TABLES

Supplementary Table 1. Detailed ingredients of NCD, HSD, and HFD.

	NCD	HSD	HFD
Product No.	D16022602	D16022604	D12492
**Ingredient (g)**			
Casein	160.5	160.5	200
DL-Methionine	3.0	3.0	0
L-Cystine	0	0	3.0
Corn starch	442.5	0	0
Maltodextrin 10	125.0	25.0	125.0
Sucrose	0	542.5	68.8
Cellulose, BW200	50.0	50.0	50.0
Corn oil	98.4	98.4	0
Soybean oil	0	0	25.0
Lard	0	0	245.0
t-Butylhydroquinone	0.02	0.02	0
Mineral mix S10022M	35.0	35.0	0
Mineral mix S10026	0	0	10.0
Vitamin mix V10037	10.0	10.0	0
Vitamin mix V10001	0	0	10.0
Choline bitartate	2.5	2.5	2.0
Dicalcium phosphate	0	0	13.0
Calcium carbonate	0	0	5.5
Potassium citrate	0	0	16.5

Abbreviations: NCD: normal-control diet; HSD: high-sucrose diet; HFD: high-fat diet.

Supplementary Table 2. Corresponding p-values to significant comparisons shown in Figure 5.

Parameter	Time-point	Comparison<br>*(A vs. B)*	Difference<br>*(B in relation to A)*	p
AUC	Week 12	NCD WT vs. HFD WT	+ 83%	<0.001
		NCD APP23 vs. HFD APP23	+ 56%	<0.001
		HSD WT vs. HFD WT	+ 59%	<0.001
		HSD APP23 vs. HFD APP23	+ 76%	0.009
		HFD WT vs. HFD APP23	- 20%	0.017
	Week 20	NCD WT vs. HFD WT	+ 104%	<0.001
		NCD APP23 vs. HFD APP23	+ 85%	<0.001
		HSD WT vs. HFD WT	+ 63%	<0.001
		HSD APP23 vs. HFD APP23	+ 51%	<0.001
		HFD WT vs. HFD APP23	- 15%	0.049
Fasting glucose	Week 12	NCD APP23 vs. HSD APP23	+ 25%	<0.001
		NCD WT vs. HFD WT	+ 26%	0.004
		NCD APP23 vs. HFD APP23	+ 30%	0.011
		HSD WT vs. HFD WT	+ 21%	0.014
	Week 20	NCD APP23 vs. HSD APP23	+ 18%	0.027
		NCD WT vs. HFD WT	+ 21%	0.002
		HSD WT vs. HFD WT	+ 19%	0.004
		NCD APP23 vs. HFD APP23	+ 35%	0.003
Fasting insulin	Week 12	NCD WT vs. HFD WT	+ 231%	0.001
		NCD APP23 vs. HFD APP23	+ 305%	0.009
		HSD WT vs. HFD WT	+ 231%	0.003
		HSD APP23 vs. HFD APP23	+ 174%	0.039
	Week 20	NCD WT vs. HFD WT	+ 522%	<0.001
		NCD APP23 vs. HFD APP23	+ 267%	<0.001
		HSD WT vs. HFD WT	+ 479%	<0.001
		HSD APP23 vs. HFD APP23	+ 288%	<0.001

Statistical tests are described in the respective figure legend. Differences are displayed as percentage in relation to the first group of the comparison (e.g. NCD WT vs HFD WT +83% means that HFD WT shows 83% more than NCD WT). Abbreviations: AUC: area under the curve.

Supplementary Table 3. Corresponding p-values to significant comparisons shown in Figure 6.

Parameter	Time-point	Comparison (*A vs. B)*	Difference *(B in relation to A)*	p
O_2 consumption	Baseline	WT vs. APP23	+ 12%	<0.001
	Week 12	NCD WT vs. NCD APP23	+ 22%	0.030
		HSD WT vs. HSD APP23	+ 15%	0.013
		HFD WT vs. HFD APP23	+ 16%	0.002
	Week 20	NCD WT vs. NCD APP23	+ 22%	0.013
		HSD WT vs. HSD APP23	+ 14%	0.057
		HFD WT vs. HFD APP23	+ 10%	<0.001
CO_2 production	Baseline	WT vs. APP23	+ 11%	<0.001
	Week 12	NCD WT vs. NCD APP23	+ 14%	<0.001
		HFD WT vs. HFD APP23	+ 14%	0.003
		NCD WT vs. HFD WT	- 10%	0.051
		NCD APP23 vs. HFD APP23	- 10%	0.003
		HSD WT vs. HFD WT	- 15%	<0.001
		HSD APP23 vs. HFD APP23	- 9%	<0.001
	Week 20	NCD WT vs. NCD APP23	+ 16%	0.018
		HFD WT vs. HFD APP23	+ 13%	<0.001
		NCD WT vs. HFD WT	- 18%	<0.001
		NCD APP23 vs. HFD APP23	- 20%	<0.001
		HSD WT vs. HFD WT	- 22%	<0.001
		HSD APP23 vs. HFD APP23	- 15%	<0.001
Respiratory exchange ratio	Week 12	HSD WT vs. HSD APP23	- 9%	0.010
		NCD WT vs. HFD WT	- 16%	<0.001
		NCD APP23 vs. HFD APP23	- 11%	0.009
		HSD WT vs. HFD WT	- 16%	<0.001
		HSD APP23 vs. HFD APP23	- 9%	0.003
	Week 20	HSD WT vs. HSD APP23	- 10%	0.020
		NCD WT vs. HSD WT	+ 7%	0.049
		NCD WT vs. HFD WT	- 16%	<0.001
		NCD APP23 vs. HFD APP23	- 14%	<0.001
		HSD WT vs. HFD WT	- 22%	<0.001
		HSD APP23 vs. HFD APP23	- 14%	<0.001
Activity	Baseline	WT vs. APP23	+ 38%	0.009
	Week 12	NCD WT vs. NCD APP23	+ 84%	0.016
		HFD WT vs. HFD APP23	+ 57%	0.061
		NCD APP23 vs. HFD APP23	- 46%	0.016
		HSD WT vs. HFD WT	- 46%	0.004
	Week 20	NCD WT vs. NCD APP23	+ 50%	0.010
		HFD WT vs. HFD APP23	+ 71%	0.007
		NCD APP23 vs. HFD APP23	- 44%	0.001
		NCD WT vs. HFD WT	- 50%	<0.001
		HSD WT vs. HFD WT	- 60%	0.007
		HSD APP23 vs. HFD APP23	- 40%	0.002

Statistical tests are described in the respective Figure legend. Differences are displayed as percentage in relation to the first group of the comparison (e.g. NCD WT vs HFD WT +83% means that HFD WT shows 83% more than NCD WT).

Supplementary Table 4. Results of spearman correlation of energy expenditure with lean mass, corresponding to Figure 6J–6L and Supplementary Figure 1J–1L, and to Supplementary Figure 2G–2I, 2P–2R.

Figure	Time-point	r_{WT}	p_{WT}	r_{APP23}	p_{APP23}
Figure 6J-L	Baseline	0.348	0.041	0.607	0.001
	Week 12	0.662	<0.001	0.673	<0.001
	Week 20	0.615	<0.001	0.550	0.003
Supplementary Figure 1J–1L	Baseline	0.511	0.002	0.696	<0.001
	Week 12	0.662	<0.001	0.769	<0.001
	Week 20	0.734	<0.001	0.816	<0.001
Supplementary Figure 2G–2I	Baseline	0.179	0.305	0.642	<0.001
	Week 12	0.503	0.002	0.653	<0.001
	Week 20	0.488	0.003	0.489	0,004
Supplementary Figure 2P–2R	Baseline	0.417	0.013	0.629	<0.001
	Week 12	0.624	<0.001	0.793	<0.001
	Week 20	0.638	<0.001	0.826	<0.001
